# On Intestinal Parasitism in the Dog, and Its Treatment

**Published:** 1896-06

**Authors:** Cecil French

**Affiliations:** D.V.S. (McGill and Munich), The Washington (D. C.) Canine Infirmary


### THE JOURNAL
OF
### COMPARATIVE MEDICINE AND VETERINARY ARCHIVES.

VOL. XVII. *JUNE*, 1896. No. 6.

### ON INTESTINAL PARASITISM IN THE DOG, AND ITS TREATMENT.

BY CECIL FRENCH, D.V.S. (MCGILL AND MUNICH),
THE WASHINGTON (D. C.) CANINE INFIRMARY.

IN former times, before the complicated life-history of intestinal parasites had become established through experimental research on men and animals, the condition known as helminthiasis was understood to indicate a certain *diathesis verminosa*,[1] the essential characteristic of which was malassimilation of nutriment, resulting in the accumulation of material which contributed to the production of worms—that their presence could be accounted for by spontaneous generation. It was held by some[2] that this worm-diathesis could exist without the actual presence of the parasites. Others even asserted that they exerted a beneficial effect by increasing secretions and stimulating peristalsis. Not only were such false ideas conceived, but it was the belief of the medical fraternity that every ailment had its origin in the existence of some real or imaginary verminous parasite, each with a characteristic habitat. Traces of this erroneous credulity exist to this day among the laity, as every practitioner knows and experiences to the cost of certain appendages of his patients. It is now well established that intestinal worms can only occur as the result of ingestion of themselves, their eggs, or their larvæ.

Of all domestic animals that come within the range of our practice none is so notoriously the harbinger of intestinal parasites as the dog, with the exception, perhaps, of the cat. In fact,

[1] Bremser. [2] Rilliet, Barthez.

so conveniently does he prove a host, through his methods of seeking and partaking of his daily aliment, that more deaths are ascribed, either directly or indirectly, to this cause alone than to all others put together.

The different species of worms produce various pathological conditions, according to the influence they exert and the part of the alimentary canal they inhabit. Thus the symptoms produced by ascarides differ considerably from those produced by uncinaria. In considering the influence exerted by these parasites on their host several factors must be entertained:

1. They exist and thrive at the expense of their host. The importance of the drain on the host of nutriment depends on the variety, number, and size of the parasites present. It may take place either by absorption of digested matter, as occurs from the presence of tape and the common round-worms; by direct extraction of blood through the attachment of uncinaria to the mucous membrane, and the subsequent secondary hemorrhages resulting from their bites; or, in slight measure, by the devouring of cells of certain parts of the tract which characterizes infection from sporozoa.

2. Their presence, and more particularly their movements, may inhibit and prevent the physiological functions of the intestine, and by irritation of the sympathetic nerve-endings produce reflex nervous phenomena of a most severe character. This latter is the most serious effect.

3. It has not been proven, but from clinical symptoms it would seem highly probable, that, like bacteria, they may produce certain leucomaïns having a decidedly injurious action, which manifests itself prominently in cutaneous eruptions.

4. Finally, they exert local influence by their bulk, in that they may form impacted masses in the canals in which they live, and may wander into and occlude neighboring passages, such as the bile-ducts and respiratory tract.

The life-history of at least the more common forms with which we have to deal should be made the object of careful study, since on this depends a knowledge of the methods employed for their prevention and eradication.

It may be briefly stated that the ascaris is most dangerous, the tænia most insidious, and the uncinaria or dochmius most difficult of expulsion. So profound is the influence on the nervous system exerted by the first, that an apparently healthy, vigorous puppy may be suddenly stricken down, perhaps with one or two convulsive movements, and succumb from reflex cardiac inhibition. The second may merely induce in the host slight periods of listlessness, which often fail to be taken as indicative of tæniasis. The last may have stimulated the secretion of such an abundance of tenacious mucus that it may envelop and protect them from the action of the remedy. Moreover, the heads of the same being attached at some depth within the wall of the mucosa, the difficulty of reaching them with remedies is further augmented.

In the treatment of intestinal parasitism we aim either to kill or torpify the worm, and then remove it from the intestine by the aid of purgatives. Remedies used for this purpose are designated vermicides or anthelmintics. Or we may drive out the parasite living, when the drugs we use would be more properly called vermifuges.

It is best to begin with the preparatory treatment. This consists in cleaning out the intestines, particularly the lower bowel, so that there may exist no obstruction to the free exit of the worm. For this purpose laxatives may be administered, and their action supplemented by an enema of cold water. All solid food should then be withheld for a day or two. It is customary with some to proceed with a special diet, the object of which is to "sicken the worm." Röll[1] recommends a preparatory diet of plenty of meat, or a decoction of milk or onions. The feeding of meat at this period is hardly to be counselled. It requires considerable time—sometimes hours—for the completion of gastric digestion in the carnivora, and especially is this the case when meat has been swallowed in large fragments. The presence in the canal of the débris of such fare must of necessity more or less impair the desired effect of any remedy, unless several hours have elapsed prior to its exhibition. Milk is an excellent preparatory food, and may be given unsparingly.

Treatment is best commenced in the morning. The patient should previously be fasted from twelve to twenty-four hours, according to age and physical condition. The older writers enjoin that the anthelmintic prescribed should be followed an hour or so later by purgatives, such as castor oil, etc. Such procedure is usually necessary if the remedies and methods of applying them then in vogue are made use of; but under our

[1] Röll: Manuel de pathologie et de thérapeutique des anim. dom.

modern system of medication, with the improved and concentrated drugs now prepared by the chemists, vermicide and purgative can be combined and administered in small capsular form, with little or no inconvenience to either practitioner or patient. While the so-called gunshot prescriptions are usually regarded as unscientific, there can be no doubt that in the majority of cases of helminthiasis, especially where a positive diagnosis of the worm present is not possible from examination of the excreta, the wisest mode of procedure is to administer remedies that will attack both tape- and round-worm at the same time, for it frequently happens that both classes are present in the canal together.

Many of our clientèle desire to tend their pets themselves during treatment for helminthiasis. They request of us compound or prescription for the same, that they may administer with a minimum of difficulty; they do not care to have to force their pets to swallow an ounce or two of objectionable and often nauseating castor oil or any other fluid substance. With this object in view I have endeavored to secure a combination of drugs with both anthelmintic and purgative properties in such form as will admit of easy administration. The prescription I use for this purpose is as follows:

| | |
|---|---|
| ℞.—Oleoresinæ Aspidii . . . . . | 5 ♏. |
| Kamalæ . . . . . . . . | 3 grains. |
| Santonini . . . . . . . . | ½ grain. |
| Resinæ Jalapæ . . . . . . | 1 grain. |
| Hydrargyri Chloridi Mite . . . . | ½ grain.—M. |

The first ingredient is rated among our most active tænicides. In addition, it has proved a valuable remedy against dochmius. The second is also a tænicide with drastic purgative properties, and has some effect on dochmius. The third is, *par excellence*, our weapon against round-worm, whilst the two last are active purgatives for the dog, and, inasmuch as the resin of jalap or jalapin is used, requires to be given in but very small dose.

Parke, Davis & Co. prepare this for me and put it up in their excellent soft elastic gelatin capsules, each containing the quantities designated in the prescription, at a cost of $5.75 per 1000 capsules. For puppies from six to ten weeks old (excepting very small toy-dogs) I prescribe one capsule, to be followed by a second in three or four hours' time, and increase the doses up to four or six capsules according to the age and breed of the dog.

Though the removal of intestinal parasites is a violent procedure, it is usually accomplished without much distress to robust subjects. Nevertheless, with weak, unthrifty, or highly nervous animals, especially during puppyhood, it is sometimes far from being unattended with dangerous or even fatal results. Particularly is this the case with ascarides. There can be little doubt that such effects have frequently been unwittingly ascribed to the systemic action of the drug or drugs used, whereas in reality they have been occasioned solely by the gyrations of the worm attending the act of removal. Tænia and dochmius exert on their host an effect that may be described as *passive*, and can be considered as seldom capable of inciting sudden danger to life. On the other hand, the effect produced by ascarides is most *active* and often precipitate.

When an ascaris has slowly developed in a certain area of the intestinal canal from its ovoid state to maturity, it no doubt remains in a more or less quiescent condition so long as it is supplied with abundant nutriment and comes in contact with nothing that will affect its equilibrium. In other words, it will adapt itself readily to the favorable environment. It is not unreasonable to suppose that a like adaptation to prevailing conditions will be gradually acquired by the division of the nervous system distributed to the area in question. So long as no profound change takes place in the relationship of the worm to itself the nervous system will continue to be unaffected. If, however, something noxious in the host's diet (and the appetite of a worm-subject is notoriously abnormal) or indisposition on the part of the host to partake of the regular supply intervene, the parasite in its struggle for existence will begin migratory action in search of a retreat or sufficient nutriment, as the case may be. Then will follow the train of nervous phenomena symptomatic of the presence of ascarides. The nervous relationship has been rudely jarred and the intensified movements of the worm exert an impressive reflex action on the higher centres.

Applying this hypothesis to the unfavorable results sometimes following the removal of the parasites we are afforded a reasonable explanation for the nervous phenomena and sudden collapse. It requires but a slight stretch of the imagination to apprehend what a profound effect violent and abnormal gyratory movements of a fugitive round-worm may exert on the nervous system. The cautious practitioner, on receiving an asthenic patient for anthelmintic treatment, accordingly will not hesitate to advise his client of a possible unfavorable termination to his efforts to effect a cure.

The following are the most approved anthelmintics for dogs, arranged as nearly as possible in their order of general efficacy. The doses mentioned range between the extremes for the smallest puppy to the largest full-grown animal:

*For Tænia.*

GRANATUM (*U. S. P.*) is official, but its use in crude form is impracticable. Its vermicidal properties depend on its alkaloid, pelletierine, which is a colorless liquid, soluble in twenty parts of water, and readily miscible with alcohol, ether, or chloroform. By the addition of acids, crystalline salts are formed, of which four have been prescribed, viz., the sulphate, hydrobromate, hydrochloride, and tannate. Tanret, in 1878, announced the successful isolation of the alkaloid pelletierine. Its use at first, in the form of the sulphate salt, was not attended with the expected favorable results. This was attributed to the decomposition of the salt by the acid of the stomach. The tannate was then employed with complete success, on account of its small solubility and slow absorption. It is a hygroscopic, tasteless powder as prepared by Merck. Pelletierine is undoubtedly the most efficient and innocuous tænicide for the dog that we possess, but is not much used on account of its expense. I have frequently found it most useful when the stomach has refused to retain other remedies. The doses of pelletierine tannate are, for adults, 5 to 15 grains; puppies, ½ to 5 grains. It should be administered in gelatin-capsular form in conjunction with powdered purgatives.

A preparation of the alkaloid known as Tanret's syrupy solution is sold, each bottle containing what must be considered a small dose for the dog. Another preparation is the *Extractum punicæ granati fluidum*, adult dose 20 to 30 minims, to be administered in syrup, but its effects are uncertain.

ASPIDIUM (*U. S. P.*), FILIX MAS (*B.P.*), is perhaps the most reliable of all the vermifuges, with the exception of pelletierine. For every-day practice it is to be preferred to all other remedies when given in the form of the oleoresin (*Oleoresina aspidii, U. S. P.*). The doses are, for adults, 15 to 40 minims; puppies, 5 to 15 minims. The dose of the (*B.P.*) liquid extract (*Extractum filicis*

*liquidum*) is the same. It is worthy of note that Fröhner reported the poisoning of a small dog by 30 minims, and a medium-sized one by 5 fluidrachms of the liquid extract. Müller regards the extract the most reliable of all tænicides.

KAMALA (*U. S. P.; B.P.*) is a brick-red powder containing an active principle, rottlerin, which is not official. It is a very efficient tænicide with drastic purgative properties. Given in small amount as an adjunct to other tænicides, particularly to the oleoresin of male-fern, it will be found a very valuable remedy. The doses are, adults, 15 to 30 grains; puppies, 3 to 15 grains.

BRAYERA (*U. S. P.*), CUSSO (*B.P.*), yields kosin or koussein, to which it owes its tænicidal properties. As prepared by Merck it is a yellowish-brown powder, soluble in alcohol and ether, but sparingly so in water. It is one of the best and safest tænicides, its action being directly toxic to the worm, but it is too expensive for ordinary practice.

The infusion (*Infusum brayeræ* (*U. S. P.*) and fluidextract (*Extractum brayeræ fluidum*) are both too bulky and disagreeable for administration to the dog. Kosin may be given in capsules in doses, adults, 10 to 40 grains; puppies, 10 to 20 grains. The drug usually acts as its own cathartic, but it is better to employ some adjunct for this purpose.

SEMEN ARECÆ, when freshly ground into powder, is a very good remedy for tapeworm, with some effect on ascarides also. When stale it will generally be found inert, consequently it is best always to purchase the nut and grind or grate on an ordinary nutmeg-grater. It is still largely used by British veterinarians, and is a favorite with some on this Continent, but it cannot now be regarded as being either as effectual or easy of administration as the two preceding drugs. Its effects on young puppies are not unattended with danger, on account of its great astringency, but with due regard to subsequent purgation it is a perfectly safe remedy.

Mayhew's method of prescribing one to two grains to every pound weight of the patient is usually followed, but the smaller quantity will generally suffice, provided the powder is freshly ground. It may be conveniently given in gelatin capsules, accompanied or followed by a purgative. Combined with the fluidextract of male-fern it is highly recommended by the Edinburgh authorities.

TEREBINTHINA (*U. S. P.*) is a powerful remedy against tapeworm, but it is regarded as being somewhat dangerous from its liability to produce strangury and renal inflammation. These effects are said to be less pronounced after large than after small doses; but large doses are more liable to cause gastric and enteric inflammations. It can hardly, therefore, rank with the best remedies. If given, it should be administered in emulsion with white of egg, mucilage, milk, or oil, in doses as follows: adults, 15 to 40 minims; puppies, 3 to 10 minims.

Dr. W. Horace Hoskins informs me that the use of this drug in his hands, where the puppies are under six months of age, has proven very satisfactory, and has never been followed by any gastric or renal trouble. In extremely young dogs he rarely gives more than two minims in milk, carrying it up to ten minims, and repeating for two or three days on an empty stomach in the morning, allowing no food for an hour or two hours after its administration.

*For Ascarides.*

SANTONINUM (*U. S. P.* and *B. P.*) is the most approved remedy against round-worm. Discovered in 1830 by Kahler, a chemist at Düsseldorf, its therapeutic properties were soon studied by Trommsdorff and Liebig. Merk, of Darmstadt, and Cailloud, of Annecy, first made known its vermifugal properties on human beings. Later, Coppola introduced santoninossima as an insoluble substitute, but there seem to be no records of its trial on dogs.

The administration of santonin to young dogs without some protective vehicle in other than relatively small doses, is not unattended with danger.

Fröhner[1] reported profound symptoms of poisoning in a puppy aged five weeks, weighing six and a half pounds. A dose of one grain was given, which had an immediate anthelmintic action. At the same time the animal manifested restlessness, ran around the room, and whined the whole night. Paralysis of the hind extremities appeared. Psychical disturbance was pronounced. The urine developed a striking reddish-yellow color. Temperature, 102.6°; pulse, 152; respirations, 62. An antidote of coffee (ʒiss to ℥iss) was administered, and recovery ensued.

A dog,[2] ten years old, weighing fifteen pounds, received gradually increasing doses of santonin, commencing with 4 grains, until ʒijss had been reached, at intervals of two to four days.

1 Monatshefte für Thierheilkunde, 1893, iv. pp. 338–309.

2 Ibid., pp. 535–563.

Not until over 75 grains had been taken did his system respond to the drug, and from then on the reaction appreciably decreased.

Keppel[1] administered 6 grains with 1½ ounces of castor oil to a dog weighing twenty pounds, increasing the dose to 15 grains in four days. The urine became of a deep-red color as the dose advanced, but there were no poisonous effects.

Another dog, weighing thirteen pounds, received 7½ grains without ill effect. In three-quarters of an hour 15½ grains were given, when epileptiform convulsions developed. The same quantity again produced the same result. Recovery followed in a few hours. V. Hasselt and Rienderhoff[2] observed the following effects from doses mentioned: 6 grains, trembling of hind extremities; 9 to 12 grains, stiffness and cramps in extremities, head, and trunk, trismus, dilatation of pupils, and unconsciousness. They regard doses of from 60 to 90 grains as fatal, death intervening after repeated attacks of cramp and profuse salivation.

The following conclusions were drawn by Fröhner with regard to the effect of the drug: Nursing puppies per two and a half pounds body-weight are nearly one hundred times, and half-grown animals two to four times, more susceptible than adults. Thus, an aged animal taking 26 grains per two and a half pounds body-weight exhibited almost complete immunity, whilst in the first-mentioned case a puppy nearly succumbed to slightly more than ⅓ grain per two and a half pounds body-weight. A three-weeks-old puppy,[3] weighing four and a half pounds, was unaffected by 4½ grains administered in glucose; 9 grains did not render the animal unconscious, but produced slight contraction and uneasiness. A medium-sized dog got 7½ grains in sugar. In three-quarters of an hour the urine became of an onion-red color, after three hours very purple, and in twenty-one hours assumed its normal color again. The same animal was given santonin in castor oil without influencing the elimination of urine.

From the foregoing cases it would seem that when the drug is suspended in some oleaginous or other suitable vehicle (oil, cream, sugar of milk), it becomes less liable to absorption on

[1] From extract from Bericht des Vet. W. in Sachsen in American Vet. Review, May, 1894, pp. 116–117.

[2] Monatshefte für Thierheilkunde, 1893, iv., pp. 308–309.

[3] From extract from Bericht des Vet. W. in Sachsen in American Vet. Review, May, 1894, pp. 116–117.

account of its being but imperfectly dissolved. Santonin is not soluble in water or weak acids, like the gastric juice. It is probable that when given alone it passes through the stomach unchanged until it meets the alkaline juices of the small intestine, in which it is freely soluble. If administered already dissolved in an alkaline menstruum, it is liable to be absorbed in the stomach before reaching the parasite. Accordingly, we are led to the conclusion that it is considerably safer to administer santonin suspended in either of the substances mentioned than by itself. But for reasons already stated it is frequently impracticable to prescribe oleaginous compounds. Pill or capsule, with sugar of milk as a vehicle, and containing at the same time a powdered purgative, meet these requirements, but as such the dose should be the smallest. But, as already stated, small doses of santonin occasionally exert but little more than vermifugal action. If the worms be present in large numbers such doses may be sufficient to incite sudden and migratory action on the part of the parasites, which, though complete as far as the desired remedial effect of the drug is concerned, may profoundly affect the nervous system in debilitated or impressionable subjects. On the other hand, doses sufficient to produce a pronounced vermicidal effect are attended with considerable risk to young subjects.

Seeing that the nervous disturbances in question are not dangerously frequent the balance of safety lies in favor of small amounts, and these should always be administered when possible suspended in either of the substances mentioned above. Theoretically, oil should be the best vehicle, because it tends to lubricate and render less susceptible to irritation the surface over which the struggling worm travels.

Not more than the doses mentioned below should be given, but they may be repeated at intervals of a few hours. The crystals should be used and not the powdered santonin—adults, 1 to 4 grains; puppies, ⅙ to ½ grain. As a purgative adjunct when prescribed in capsule, calomel is regarded as particularly useful, the outpouring of bile caused by the mercurial being abhorrent to the worm.

Fröhner[1] regards doses of ⅓ grain per two and a half pounds body-weight liable to induce severe poisoning in young dogs, and suggests as a safe dose for puppies ⅙ to ⅓ grain, for old

[1] Monatshefte für Thierheilkunde, 1893, iv. pp. 308, 309.

dogs ⅔ to 3 grains. Poisonous but not necessarily fatal doses are 1¼ to 2½ drachms. However, grown animals in advanced years so accustom themselves to the drug that it is difficult to poison them with it. Therapeutic doses can in general be much larger for grown animals than for young ones. Doses which may be regarded as large but not always dangerous for grown animals are 7 to 30 grains. With these doses the animals in Fröhner's experiments showed only slight if any reaction. The principal symptoms of poisoning from santonin in the dog are epileptiform convulsions, psychical aberration, lethargy, vertigo, paralysis, disturbance of vision, myosis, yellow-red coloration of urine, salivation, nasal discharges, slight colicky pains, diarrhœa, anorexia. The red coloration of urine can be seen within an hour after administration of the drug.

SPIGELIA (*U. S. P.*) is a good remedy, but not nearly as efficient as the preceding. In the form of the fluidextract (*Extractum spigeliæ fluidum* (*U. S. P.*), or, better still, the unofficial fluidextract of spigelia and senna (*Extractum spigeliæ et sennæ fluidum*). When administered without the senna it should be followed by a purge to sweep out the narcotized worm. For this purpose sulphate of magnesium is best on account of its rapidity of action. The doses of both extracts are the same: adults, ½ to 2 fluidrachms; puppies, 10 to 30 minims.

CHENOPODIUM (*U. S. P.*). The powdered seeds of this fruit, made into an electuary with syrup or honey, or enclosed in capsule, may be given in the following doses: adults, ½ to 1 drachm; puppies, 10 to 30 grains. Or it may be given in the form of the oil (*Oleum chenopodii*, *U. S. P.*). Dose, adults, 20 to 40 minims; puppies, 5 to 10 minims. To a puppy the latter should be given in emulsion with gum-acacia, but adults will bear it in capsule.

### *For Uncinaria.*

For these tenacious parasites I have found the oleoresin of male-fern combined with kamala very effectual. The former has been used with excellent results in the form of the ethereal extract by Perroncito. The latter is recommended by Mégnin,[1] in conjunction with calomel and the oxide of arsenic. The doses should be somewhat smaller than those indicated for tæniasis, and repeatedly administered. Thymol has been successfully prescribed and regarded as a specific for the closely related

[1] Traité des Maladies Parasitaires des Anim. Domest. Neumann, p. 453.

anchylostomum duodenale of the human family by certain Italian physicians, but in my hands it has failed to have any effect on the species infecting the dog, principally on account of its tendency to produce emesis, which no doubt takes place before it reaches the bowel.

Whatever remedy is used it is important that treatment be continued till all indications of the presence of these parasites have disappeared. Resort should then be had to tonics and hæmatinics with cod-liver oil.

Digestion in the stomach must be more or less impeded because the glands will secrete less vigorously during the anemic condition of the blood. Absorption of digested material from the intestine must also be considerably interfered with on account of the catarrh and inflammatory thickening present. It is important, therefore, that as much nourishment be extracted from the food and absorbed within the stomach as possible. To this end digestant doses of pepsin and acid with the food are beneficial, but should not be kept up long enough to interfere with the natural secretions.

